# SEOM clinical guideline for treatment of cancer pain (2017)

**DOI:** 10.1007/s12094-017-1791-2

**Published:** 2017-11-10

**Authors:** C. Jara, S. del Barco, C. Grávalos, S. Hoyos, B. Hernández, M. Muñoz, T. Quintanar, J. A. Meana, C. Rodriguez, R. de las Peñas

**Affiliations:** 1 Hospital Universitario Fundación Alcorcón, Universidad Rey Juan Carlos, Madrid, Spain; 2 Hospital Universitario Dr. Josep Trueta (ICO), Gerona, Spain; 3 Hospital Universitario, 12 de Octubre, Madrid, Spain; 4 Hospital Rey Juan Carlos de Móstoles, Madrid, Spain; 5 Complejo Hospitalario de Navarra, Pamplona, Spain; 6 Hospital Virgen de la Luz, Cuenca, Spain; 7 Hospital General Universitario de Elche y Vega Baja, Alicante, Spain; 8 Hospital General Universitario de Alicante, Alicante, Spain; 9 Hospital Universitario de Salamanca, Salamanca, Spain; 10 Consorcio Hospitalario Provincial de Castellón, Castellón de la Plana, Spain

**Keywords:** Cancer pain, Neuropathic pain, Breakthrough pain, Opioids, Coanalgesics

## Abstract

Pain is a highly prevalent symptom in patients with cancer. Despite therapeutic advances and well-accepted treatment guidelines, a percentage of patients with pain are under-treated. Currently, it has been recognized that several barriers in pain management still exist and, in addition, there are new challenges surrounding complex subtypes of pain, such as breakthrough and neuropathic pain, requiring further reviews and recommendations. This is an update of the guide our society previously published and represents the continued commitment of SEOM to move forward and improve supportive care of cancer patients.

## Introduction

Pain is one of the most common symptoms related with cancer and its treatment [1]. Prevalence ranges from 39% in patients following curative treatment up to 66–80% in advanced or terminal phases [2]. Several epidemiological studies carried out in Spain have shown that approximately 55% of cancer patients suffer from pain [3]. Of these, 20–33% display neuropathic pain [4], and breakthrough cancer pain is present in 41% [5].

Specific cancer types such as pancreatic, primary bone, lung and head and neck cancer associate particularly high prevalence rates of neuropathic pain, while bone metastasis is the most common cause of cancer-related pain [6]. Moreover, it changes over time along the course of disease and is most frequent in late phases of the oncologic process.

Despite the tremendous progress in the knowledge about cancer-related pain and its treatment, recent studies have shown that pain is not adequately controlled in up to 31% of cases [7]. Several barriers to adequate pain management in patients with cancer have been acknowledged: lack of knowledge among health professionals regarding cancer pain assessment and management; fear of the adverse effects of opioids; patients struggle with misconceptions about analgesic use, and concerns surrounding pain communication [8]. We must overcome obstacles and develop and implement interventions to manage pain optimally in patients with cancer. Medication should not be the sole approach; educational interventions for patients and professionals can contribute to successfully managing pain [9].

Regular, adequate, self-report assessments of pain intensity with the help of validated multidimensional assessment tools are needed for effective treatment. We must work towards a pain assessment approach that can both accurately diagnose and monitor a patient’s specific pain, while still being simple enough to be used in routine clinical practice [10]. Whenever possible, patients should be encouraged to be active participants in the management of their own pain [11]. Their caregivers should also be given and taught to use a pain diary to monitor all pain concerns. The use of technological advances may improve the accuracy of patient-reported outcomes [12].

The purpose of this document was to establish recommendations that can be applied by professionals in their clinical practice to optimize cancer pain management.

### Guideline methods

Under the auspices of the Spanish Society of Medical Oncology (SEOM), a number of experts in the field, together with two coordinators, were designated to draft these evidence-based, clinical practice guidelines. The recommendations and evidence have been graded, based on the guideline development recommendations [13].

## First and second step

### Mild pain (first WHO analgesic step)

Non-opioids, such as paracetamol and NSAIDs, must be considered for management of cancer pain in this setting. They are useful in mild or mild/moderate pain and there is no evidence to claim that some NSAIDs are more effective or safer than others [14]. At therapeutic doses, all of them present anti-inflammatory, analgesic, and antipyretic properties to a greater or lesser extent. Paracetamol and NSAIDs are effective drugs at any step of the WHO analgesic ladder, regardless of their intensity and provided that their use is not contraindicated (level of evidence I, degree of recommendation A). Some studies have reported that the combination of paracetamol with stronger opioids improves pain management and increases the sense of wellbeing [15]. Adverse effects of NSAIDs include gastrointestinal, renal, hematologic, and pulmonary effects. It is recommended that a limited number of drugs be used, depending on the clinician’s expertise and keeping patient’s references/tolerance in mind.

Combining two NSAIDs does not improve analgesia and increases toxicity.

NSAIDs and paracetamol do not cause tolerance but do have a therapeutic ceiling and used above the maximum recommended dose, do not increase the analgesic effect; however, they do increase toxicity.

### Moderate pain (second WHO analgesic step (VAS 3–6/10)

After assessing the individual, analgesic treatment is selected according to their VAS score [16]. Mild opioids are the basis of treatment (in combination or not with drugs described in the first step). Step 2 includes: codeine, dihydrocodeine, and tramadol. All of these compounds are available in controlled-release forms. Low doses of transdermal fentanyl and buprenorphine can also be considered [16]. Some studies have shown that effectiveness at the second step of the WHO ladder lasts for about 1 month for most patients, due to insufficient analgesia. Since weak opioids have therapeutic ceiling, some authors have proposed abandoning up their use in moderate pain in favor of early initiation of the third step with low doses of strong opioids [16].

Mild opioids could be prescribed in combination with non-opioid analgesics (level of evidence III, degree of recommendation C). However, in a meta-analysis of randomized clinical trials, no significant difference was found between 1st step analgesics alone and combining them with a mild opioid [16]. Their effectiveness is limited with time (40 days) and due to their dose ceiling effect, above which there is no additional analgesic effect, but an increase in side effects (level of evidence I, degree of recommendation B) [17].

Low doses of strong opioids together with non-opioid drugs can be weighed as an alternative to mild opioids (*level of evidence III, degree of recommendation C*).

### Severe pain (third WHO analgesic step (VAS > 6/10)

Neuropeptides such as enkephalins, dynorphins, and endorphins interact at opioid receptors (MOP; DOP; KOP; NOP) located in the CNS, pituitary, and GI tract [18]. Opioid agonists lock onto receptors, blocking neurotransmitters release. Opioid agonists lock onto receptors, blocking the release of neurotransmitters. Opioids differ in affinity, pharmacokinetics, physicochemical properties, side-effect profiles, administration routes, tolerance, and immunomodulation propensity [11]. Opioid antagonists bind to opioid receptors but produce no analgesia.

Strong opioids are the cornerstone of analgesia in this setting. Morphine, methadone, oxycodone, hydromorphone, fentanyl, and buprenorphine are the most widely used in Europe [12].

Opioids undergo metabolism in the liver: phase I—via CYP450; phase II—glucuronidation via UGT [19]. Age, genetics, comorbidities, kidney or liver function, and concomitant drugs can affect their metabolism; consequently, the choice of one over another must factor in these factors.

The available evidence suggests that oral morphine, hydromorphone, oxycodone, and methadone provide similar efficacy (level of evidence I, degree of recommendation A) [20]. The choice should take into account efficacy, safety, and flexibility (*level of evidence II, degree of recommendation B*). Morphine is the gold standard, given its versatility (oral, rectal, s.c., i.v., i.m., intrathecal routes), safety, and price (Table 1). The first choice is oral morphine (*level of evidence IV, degree of recommendation D*) [21, 22]. When urgent relief is required, titrate with parenteral opioids [12]; likewise, they may also be used in patients for whom oral opioids are not suitable and analgesic requirements are unstable. The equianalgesic ratio between oral and parenteral routes is 2:1 or 3:1 (*level of evidence II, degree of recommendation A*).

**Table 1:** WHO 3rd step Adapted from Ripamonti C. ESMO Guideline [16]

Drug	Route	Relative effectiveness compared to oral morphine	Maximal daily dose (the maximal dose depends on tachyphylaxis)	Starting dose in opioid-naïve patients
Morphine sulfate	Oral	1	No upper limit	20–40 mg
Morphine	i.v. (s.c.)	3	No upper limit	5–10 mg
Fentanyl transdermal	TTS	+ 4	No upper limit	12 mcg/h
Methadone	Oral	4 - 8- 12 (Factors corresponding to daily morphine doses < 90, 90–300 or > 300)	No upper limit	10 mg

Transdermal (TTS) opioids (fentanyl, buprenorphine) are valid alternatives when oral opioids are not suitable and analgesic requirements are stable (*level of evidence II, degree of recommendation A*). TTS fentanyl displays good patient compliance *(level of evidence I, degree of recommendation B).* There is insufficient evidence as yet to support the use of combination opioid therapy. Strong opioids can be combined with continued use of a non-opioid analgesic (*level of evidence II, degree of recommendation B*). In renal impairment opioids should be used with caution; in this setting, buprenorphine is the safest (level of evidence IV, degree of recommendation C) [23].

Titration is the process by which a tailored dose is found that provides pain relief with lowest possible degree of toxicity. Normal release opioids are indicated for titration and treating breakthrough pain episodes. All patients should receive round-the-clock dosing and a ‘breakthrough dose’ (usually 10–15% of the total daily dose) to manage breakthrough pain (*level of evidence IV, degree of recommendation C*). Once the appropriate dose has been determined by means of titration, slow-release opioids are indicated (*level of evidence IV, degree of recommendation C*). Other recommendations: respect patients’ preferences whenever feasible; correct myths and misconceptions; ensure the patient has accurate information so as to improve compliance [21].

### Management of side effects

The main toxicities associated with opioids consist of: GI (constipation, nausea, vomiting), CNS (cognitive impairment, hyperalgesia, allodynia, and myoclonia), respiratory depression, and others (pruritus, dry mouth, urinary retention, hypogonadism, and immune depression) [24].

Management includes the following: (1) patient information and prophylactic measures; (2) reduction in opioid dose through the use of a co-adjuvant and/or first step drug; (3) pharmacological strategies, such as antiemetics for nausea, laxatives for constipation, tranquillizers for confusion, psychostimulants for drowsiness, and (4) switching to another opioid or route. For persistent constipation, consider PAMORAs (peripherally acting mu-opioid receptor antagonists) with demonstrated benefit in non-oncological settings. Naloxegol per os was approved by the EMA for opioid-induced constipation in adult cancer patients. Naloxone is an antagonist capable of reverting symptoms of severe opioid overdose.

### New opioids

New opioids have been developed in recent decades with different metabolic pathways, delivery systems, or receptor activities [25]. Several systematic reviews support the use of oral morphine, oxycodone, or hydromorphone for cancer pain. No differences between analgesic efficacy and tolerability were found between these opioids. Consequently, oral formulations of any of these drugs can be chosen to begin step 3 for moderate to severe cancer pain (*level of evidence I, degree of recommendation A*). The choice of an opioid should be informed by the individual’s condition and clinical need.

**Oxycodone** is a semisynthetic derivative of thebaine and, therefore, does not undergo the same metabolic changes as morphine. This makes it especially useful in the elderly, as well as patients presenting impaired hepatic/renal function or comorbidities. Both long-acting and short-acting presentations are available. Naloxone is a competitive antagonist of opioid receptors and acts on bowel transit via different mechanisms, avoiding opioid-induced constipation. The combination of oxycodone and naloxone up to a dose of 160/80 mg per day is effective and generally well tolerated **(** *level of evidence I, degree of recommendation B*) [26].

**Hydrocodone** has been marketed with acetaminophen presentations which precludes the use of higher doses. It is metabolized at the liver involving cytochrome P450. Drug inhibiting CYP3A4 activity may result in increase plasma concentration of the drug. **Hydromorphone** is the active metabolite of hydrocodone; is more potent and has a higher affinity for μ-opioid receptor, and is eliminated by the kidneys. It has a short half-life; consequently, it can be used to titrate doses. Its long-acting presentation has the advantage of being taken once daily. The latest review involved 604 patients from four trials that compared hydromorphone to oxycodone or morphine. Similar analgesic efficacy was demonstrated between groups with both comparisons (*level of evidence II, degree of recommendation A*) [27].

**Tapentadol** is a centrally acting oral analgesic that possesses a combined mechanism of action: it is a μ-receptor agonist and norepinephrine reuptake inhibitor. Morphine milligram equivalents are based on the degree of μ-receptor agonist activity, although it has yet to be elucidated whether this drug is associated with overdose in the same dose-dependent manner as observed with medications that are solely opiate-μ-receptor agonists. Tapentadol’s antihyperalgesic effects could be potentially helpful in states of hyperexcitation, such as those observed in patients who have undergone multiple unsuccessful trials of opioids [28]. In contrast, a recent review in cancer patients failed to clearly demonstrate tapentadol’s superiority with respect to earlier generation opioids. Likewise, there is an absence of non-inferiority trials comparing tapentadol to fentanyl or oxycodone–naloxone [29]. As a result, tapentadol is an effective, well-tolerated alternative for moderate or severe cancer pain (*level of evidence II, degree of recommendation A*).

**Transdermal fentanyl and buprenorphine** are alternatives to morphine and may even be the preferred step III opioid for some patients **(** *level of evidence II, degree of recommendation A*). For individuals who are unable to swallow, they comprise an effective, non-invasive means of opioid delivery [30].

**Methadone** is a synthetic opioid that poses certain challenges as regards dose titration and is proven to cause potentially fatal arrhythmias in some patients. Its major disadvantage is that it has a long, unpredictable, half-life with interindividual variability as to its half-life, potency, and duration, making it more difficult to manage. It is recommended that it be initiated only by experienced practitioners (*level of evidence I, degree of recommendation B*). Given that it is lipophilic, it crosses the blood–brain barrier. In a recent update review, somnolence was found to be more common with methadone versus morphine in patients with cancer (*level of evidence II, degree of recommendation A*) [31].

## Opioid rotation

**Fig. 1:**
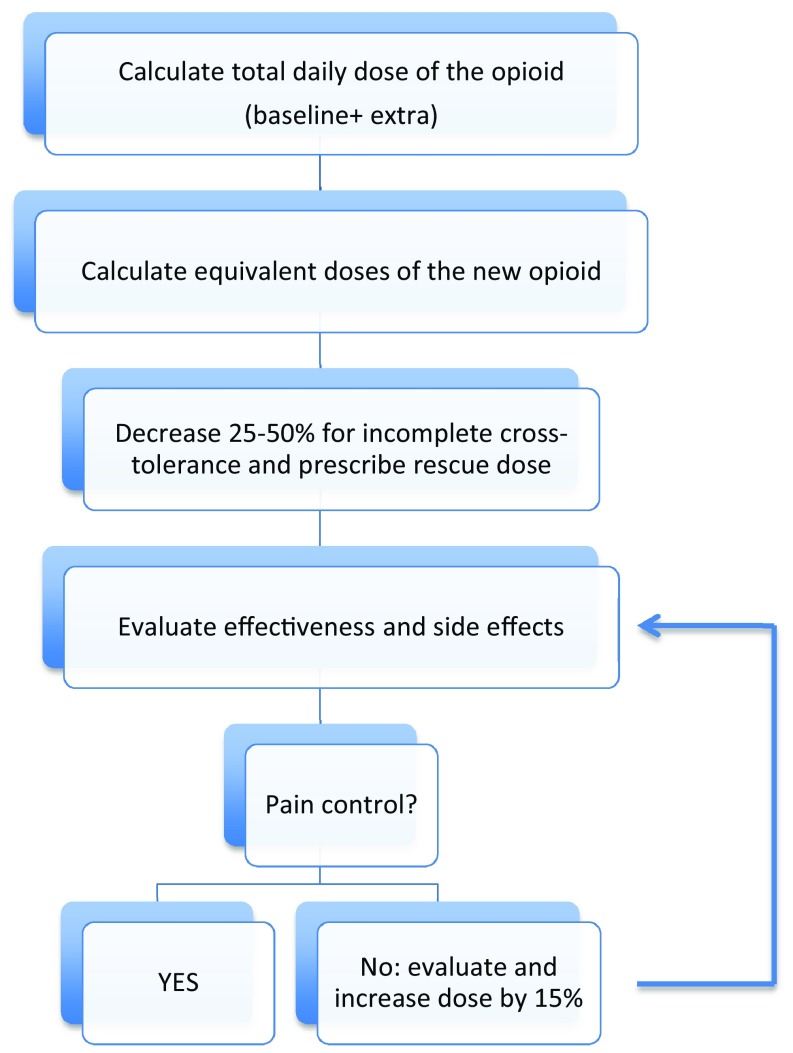
Flowchart on the rotation of opiates

Patients with cancer who experience pain often require changes in opioid therapy during the course of disease because of disease progression, pain characteristics, and prolonged use of opioids. Opioid rotation is defined as the substitution of a potent, previously prescribed opioid for a potent alternative opioid with the specific objective of obtaining a better analgesia and /or reducing unacceptable toxicity. The practice of opioid rotation is often successful (Fig. 1) although the scientific evidence remains poor because of a lack of controlled studies (*level of evidence II, degree of recommendation A*). The goal of equianalgesic rotation is to obtain the amount of opioid in the new prescription that equals the amount administered in the previous form of administration, to avoid over- or under-dosing.

Some of the most important precautions in calculating morphine milligram equivalent doses (MME) are as follows: [32].

- Equianalgesic dose conversions do not take individual variability into account.
- When calculating a new opioid, it should be dosed at a lower dose than the calculated MME to prevent overdose due to incomplete cross-tolerance and individual variability in opioid pharmacokinetics.
- The conversion factor with methadone increases at higher doses.
- Care must be exercised when converting to fentanyl or vice versa as it is dosed in μg/h.

Opioid rotation should be avoided if experience is not available or patient follow-up cannot be adequately monitored [33]. Finally, all potent opioids may have the same side effects, some of which are serious, such as respiratory depression or delirium. Naloxone (sc/iv) is reserved for the former, while delirium, myoclonus, or agitation is treated with benzodiazepines and/or neuroleptics. (Morphine milligram equivalent doses (MME) MME and some clinical situations are summarized in Table 2 [34].

**Table 2:** Morphine miligram equivalent doses (MME) and recommendations in some clinical situations Adapted from Gonzalez–Barboteo [34]

Opioid	Dosage/route	Ratio oral morphine: opioid	Recommendations
Morphine (mg)			Caution in mild to moderate renal impairment (∞)
/24 h oral	30		Caution in moderate to severe hepatic impairment
/24 h scut	15	2:1 (÷2)	Not recommended in bowel obstruction/persistent constipation
/24 h iv	10	3:1 (÷3)	DRUG OF CHOICE in case of dyspnoea, cough
Oxicodone (mg) /24 h oral	15	2:1 (÷2)	Caution in moderate to severe renal impairment (∞) Caution in moderate to severe hepatic impairment
Hidromorphone (mg) /24 h oral	6	5:1 (÷5)	Caution in mild to moderate renal impairment (∞) Caution in moderate to severe hepatic impairment Not recommended in bowel obstruction/persistent constipation
Tapentadol (mg) /24 h oral	75	1:2.5 (×2.5)	Not recommended in severe renal impairment Caution in moderate to severe hepatic impairment Caution with concomitant use of mirtazapine and antidepressants
Fentanyl (μg/h) dose/h c/72 h TTS /24 h iv or scut	12.5 300	Morphine 1 mg: 10 μg Fentanyl (×10)	Can be used in case of renal failure without dose adjustment Can be used in hepatic impairment Can be used in bowel obstruction/persistent constipation DRUG OF CHOICE in dyspnoea (in case morphine contraindicated)
Buprenorphine (μg/h) /72 h TTS	17	Morphine 1 mg: 13.3 μg Buprenorphine (×13.3)	Can be used in case of renal failure without dose adjustment Caution in moderate to severe hepatic impairment Unlike other opioids, did not present immunosuppressive activity
Methadone (mg) /8 h oral	3	< 90 mg → 4:1 90–300 mg → 8:1 > 300 mg → 12:1	Can be used in case of renal failure without dose adjustment Can be used in hepatic impairment Can be used in bowel obstruction/persistent constipation It causes prolongation QT interval DRUG OF CHOICE in cough (in case morphine contraindicated) Caution in older patients
/24 h iv or scut Continuous infusion	7		

Conversion ratios should be considered approximate. TTS: transdermal; (∞): The quality of the existing evidence on opioid treatment in cancer patients with renal impairment is low (CEBM 2a)

## Adjuvant therapy

Adjuvant therapy consists of drugs that are not primarily used as analgesics, but that possess analgesic or additive properties to opioid analgesia. Therefore, they reduce opiate doses, as well as their adverse effects, and can be used at any stage of the analgesic ladder (*level of evidence IV, degree of recommendation C*) [35]. Table 3 shows the most used as well as their main indications in pain management [35–37].

**Table 3:** Adjuvant therapies and clinical uses

Drugs	Clinical use
Antidepressants Amitriptyline, nortriptyline, imipramine, desipramine	Neuropathic pain
Anticonvulsants Pregrabalin, carbamazepine, phenytoin	Neuropathic pain
N-methyl-**d**-aspartate receptor antagonist Ketamine (+)	Pain opioid resistant Neuropathic pain
Benzodiazepines Diazepam	Muscle spasm Sleep disturbances, Anxiety
Biphosfonates Zoledronic acid, Pamidronate Calcitonin Rank inhibitor Denosumab	Bone metastasis pain
Cannabinoids (+)	Chronic neuropathic pain
Local anesthetics Lidocaine patches	Local neuropathic pain
Muscle relaxants Baclofen	Neuropathic pain
Corticosteroids Dexamethasone, Methylprednisolone, Prednisone	Spinal cord compression, Nausea, anorexia, asthenia, Pain with inflammatory component
Neuroleptics and Psychostimulants	Sleep disturbances

(+) Ketamin: no clinically relevant benefit in relieving pain or reducing opioid consumption [36]

(++) Cannabis: contradictory evidence [37]

## Breakthrough cancer pain (BTCP): evaluation and management

Although there is no universally accepted definition of BTCP, most authors have defined it as a transient exacerbation of pain that occurs either spontaneously or in relation to a predictable or unpredictable trigger, despite stable, controlled background pain [38]. BTCP is different from background pain; hence, its treatment is also different. Other terms such as ‘episodic’ or ‘transient’ pain have been used to describe BTCP, but these must not be incorrectly used for episodes of pain in a patient without adequate control of background pain, or pain just prior to their next dose (‘end-of-dose failure’) [39]. The clinical features of BTCP vary from one individual to the next, from one episode to the next, and within the same individual over time. Generally speaking, an episode of BTCP is characterized by:

Location: typically the same as background pain,

Severity: usually more severe than the background pain,

Rapid onset: maximum severity within 3–5 min,

Short duration: 15–30 min or shorter, and

Number of episodes: 3–4 per day.

Prevalence rates vary widely (35–95%), depending on the definition used and the populations studied (hospitalized or ambulatory patients, end-of-life care). BTCP can be caused by the neoplasm (70–80%), anticancer treatment (10–20%), or be unrelated to the tumor or its management (< 10%). In only one half of all episodes of pain is it possible to identify triggering factors.

Diagnosis is based on medical history, physical examination, and performance of complementary tests. Davies algorithm [38] is useful to establish diagnosis of BTCP and to discriminate it from uncontrolled background pain. A validated tool for BTCP has not yet been developed for clinical use, although there is a minimum of information that should be included in the medical history (*level of evidence IV, degree of recommendation D*) [40]

- Number of episodes;
- features of the pain: onset, duration, intensity, frequency, site, quality, and radiation;
- exacerbation and/or relief factors;
- response to analgesics or to other interventions;
- associated symptoms, and
- interference with activities of daily living.

The aim of BTCP management is to minimize the intensity and severity of each pain episode, as well as to lessen its impact on patients’ quality of life. The strategy for dealing with BTCP should be individualized, depending on a variety of pain-related (etiology, physiopathology, clinical features) and patient-related factors (performance status, disease stage, personal preferences).

An interdisciplinary approach should be considered to improve BTCP progression:

*Lifestyle changes* Reduce activities that precipitate BTCP; use special aids for daily activities or specific exercises can improve BTCP.

*Management of reversible causes* Minimize mobilization in the case of bone metastases; antitussives if cough, or laxatives if constipation.

*Modification of disease processes* Treat the underlying cause of the pain; options include surgery, radiotherapy, chemotherapy, hormone therapy, targeted therapy, and immunotherapy.

*Pharmacological management* [41] It is important to optimize background analgesia (adjuvant drugs, opioid titration, opioid switching) and supplement with rescue medication. Opioids are the drug rescue of choice for BTCP and the ideal medication should meet the following:

High potency analgesia,

rapid onset of action,

short duration of action,

minimal side effects, and

easy administration (self-administration).

Traditionally, immediate-release morphine has been used to treat BTCP, but its mechanism is not suited for this purpose. Rapid-onset opioids (ROOs) have been developed for this purpose; in particular, transmucosal and intranasal fentanyl (*level of evidence IV, degree of recommendation C*). Table 4 displays the ROOs available in Spain and their characteristics. Fentanyl should be titrated to establish the appropriate dose for each individual. Response should be monitored and side effects treated.

**Table 4:** Characteristic of ROOs Adapted from Virizuela et al [41]

Fentanyl application	Time of application	Time to onset of analgesia	Titration
Oral transmucosal applicator (Actiq^®^)	15 min	15 min	Starting dose: 200 mcg. If analgesia is not obtained within 30 min, a second unit of the same strength may be consumed. The rescue dose for the next pain episode would be 400 mcg
Oral transmucosal tablet (Effentora^®^)	14–25 min	10 min	Starting dose: 100 mcg. If analgesia is not obtained within 30 min, a second unit of the same strength may be consumed. The rescue dose for the next pain episode would be 200 mcg
Sublingual tablet			
(Abstral^®^)	Inmediate	10 min	Starting dose: 100 mcg. If analgesia is not obtained within 15–30 min, a second unit of the same strength may be consumed. The rescue dose for the next pain episode would be 200 mcg
(Avaric^®^)	Inmediate	6 min	Starting dose: 133 mcg. If analgesia is not obtained within 15–30 min, a second unit of 133 or 67 mcg may be consumed. The rescue dose for the next pain episode would be 267 mcg
Fentanyl buccal soluble film (Breakyl^®^)	15–30 min	10 min	Starting dose: 200 mcg. If analgesia is not obtained within 30 min, a second unit of the same strength may be consumed. The rescue dose for the next pain episode would be 400 mcg
Nasal sprays			
(Pecfent^®^)	Inmediate	3–5 min	Starting dose: 100 mcg. If analgesia is not obtained within 30 min, a second unit of the same strength may be consumed. The rescue dose for the next pain episode would be 200 mcg
(Instanyl^®^)	Inmediate	4–11 min	Starting dose: 50 mcg. If analgesia is not obtained within 10 min, a second unit of the same strength may be consumed. The rescue dose for the next pain episode would be 100 mcg

Data taken from summary of product characteristics

Some patients fail to achieve adequate analgesia despite correct assessment and may benefit from interventional anesthetic techniques (*level of evidence III, degree of recommendation C*).

## Neuropathic cancer pain (NCP)

NCP results from injury to the peripheral or central nervous system as a consequence of compression by or infiltration of the tumor or from treatment toxicity. Neuropathic pain is usually described as burning, numbing, or electrical, and can present with additional neurological manifestations, such as sensory changes, muscle weakness, or autonomic dysfunction. The overall prevalence of NCP varies from 5 to 40% depending on the specific patient population or if mixed pain is included [42]. Proper NCP management is complex, given the heterogeneity of etiologies, variability of symptoms, and the underlying neurogenic pathophysiology. Treatment must be tailored to each individual, initiating one medication at a time and slowly titrating to match response and tolerability [43]. NCP can be relieved by multimodal treatment following WHO guidelines. It must be remembered that most cancer patients suffer multiple types of pain [44]; nevertheless, adjuvant analgesics are proposed as first choice in purely NCP.

### Opioids for NCP

Opioids are first-line treatment for moderate to severe NCP (*level of evidence II, degree of recommendation B*). No proved difference between various opioids exists, though higher doses are usually required. A combination of adjuvant analgesics is recommended for patients displaying incomplete response. Transdermal fentanyl should not be used as first line when pain can be stabilized with other opioids (*level of evidence II, degree of recommendation B*). Tapentadol does not offer any benefit over other opioids (*level of evidence III, degree of recommendation C*) **[** 45].

### Adjuvants analgesics for NCP

In this setting, a variety of medications with analgesic properties can be used in some painful conditions, including anticonvulsants (e.g., gabapentin, pregabalin), antidepressants (e.g., SSRIs, SNRIs-duloxetine, venlafaxine, tricyclic antidepressants), NMDA antagonist (ketamine), corticosteroids, and local anesthetics/topical agents (e.g., topical lidocaine patch) (Table 5) (*level of evidence III, degree of recommendation C*) [46]

**Table 5:** Most used adjuvant in NCP

Agent	Dose	Common side effects	Precautions	LOE/GOR
Gabapentin	300–1200 mg tid	Sedation, dizziness, edema	Renal insufficiency	IIB
Pregabalin	700–300 mg bid	Sedation, dizzeness, edema	Renal insufficiency	IIC
Duloxetine	60–90 mg qd	Sedation,nausea, constipation	Hepatic dysfunction Renal insufficiency	IIB CIPN
Venlafaxine	75–150 mg qd	Nausea, dizziness, somnolence	Hepatic dysfunction Renal insufficiency	IIB CIPN
Amytriptyline	10–150 mg qd	Sedation, dry mouth, constipation, dizziness, urinary retention	Cardiac disease, glaucoma	IIB
Nortriptiline	10–150 mg qd	Dry mouth, constipation, dizziness, urinary retention	Cardiac disease, glaucoma	IIC
Topical lidocaine	1–3 patches daily	No	None	VC Allodynia

*LOE*/*GOR* level of evidence/grade of recommendation

*CIPN* chemotherapy-induced peripheral neuropathy

Gabapentin appears to have a mild to moderate benefit, but no definitive conclusion can be drawn due to the tremendous variability of study design [47]. A systematic review was unable to draw definitive conclusions regarding pregabalin’s effect in NCP (*level of evidence III, degree of recommendation C*) [48]. Two phase-3 trials of venlafaxine [49] and duloxetine [50], demonstrated decreased pain intensity in patients with NCP, although this corresponded only to a reduction of just over 1 on a 0–10 Likert pain scale (*level of evidence III, degree of recommendation C*).

## Other treatments to control cancer pain

### Interventional therapies

Despite management pain according the WHO ladder, between 10 and 20% of patients may have poorly controlled pain or may not tolerate analgesics [51]. Interventional techniques make it possible to control pain and decrease systemic analgesic [52]. These therapies typically involve modifying nerve conduction and may be non-destructive (reversible agent by injection or catheter placement) or destructive (chemical or physical methods) [53]. Patient prognosis should be considered. The main indications are failure to achieve adequate analgesia or intolerable adverse effects and pain likely to be relieved with nerve block. Following successful treatment, patients should be closely monitored with scheduled opioid reduction to avoid opiate-related side effects. Some patients with refractory pain or who are intolerant to opioids can benefit from interventional therapies, such as celiac plexus neurolysis (*level of evidence II, degree of recommendation B*) [54].

### Peripheral nerve blocks

Peripheral nerve blocks are performed to denervate specific areas and can be helpful for perioperative or acute cancer pain (pathological rib fracture or vascular occlusion). They include paravertebral or intercostal blocks, brachial plexus block, or Gassesian ganglion block (for intractable facial pain).

### Autonomic nerve bocks

In the sympathetic nervous system, afferent nerve fibers carry pain from the viscera; blocking these nerve fibers can reduce pain. Celiac plexus neurolysis improves pain relief from pancreatic cancer [55] and is commonly recommended after failure of opioid therapy; nevertheless, when distant disease is evident, success rates decrease [56]. Superior hypogastric plexus neurolysis and ganglion impar block may relief visceral pelvic and perineal pain, respectively.

### Neuroaxial infusion

Infusion of drugs into the epidural/intrathecal space leads to decrease opioid consumption (approximately 1% of opioid oral dose for intrathecal infusion) and should be considered for patients who are refractory/intolerant to systemic treatment (*level of evidence II, degree of recommendation B*). Opioid, local anesthetic, clonidine (for neuropatic pain) or ziconotide are common used as percutaneous lines or fully implanted pumps.

### Vertebroplasty/kyphoplasty

Ostelytic involvement of the spine may cause pain due to pathologically vertebral fracture and percutaneous injection of bone cement can stabilize the fractured vertebrae and relief persistent or refractory pain (*level of evidence III, degree of recommendation B*) [57]. The main specific contraindications are epidural involvement, retropulsion of bone fragments into the spinal cord or neurological damage.

### Radiation therapy (RT)

All patients with painful bone metastases should be evaluated for RT since it provides excellent and often rapid pain relief (*level of evidence I, degree of recommendation A*) [58]. Although different regimens can be used (10 × 300 cGy, 6 × 400 cGy, 5 × 400 cGy), pain relief is equivalence and ASTRO support treatment with single 8 Gy fraction based on better convenience, cost-effective and no increase toxicity (*level of evidence I, degree of recommendation A*) [59]. Reirradiation may be necessary when pain relapses, but may not be feasible due to the limited tolerance of tissues [60].

### Radiofrequency ablation for bone lesions

Nonsurgical ablation of painful skeletal metastases is possible when moderate/severe pain persists after RT. This pain is generally from osteolytic metastases that must be separable from critical structures [61]. Radiofrequency ablation relies upon a needle electrode to deliver a current that results in frictional heating (coagulative necrosis). Cryoablation provides immediate cooling that results in a visible ice ball. Focused ultrasound is a non-invasive, MRI-guided, ablative method in which energy is directed at the focal point, raising the temperature and producing thermal tissue destruction (*level of evidence III, degree of recommendation C*) [62].

### Tanezumab

Tanezumab is a monoclonal antibody that inhibits neurotrophin nerve growth factor and has been shown to reduce osteoarthritis and chronic low back pain [63]. In cancer pain, two phase-2 studies have assessed tanezumab in painful bone metastases [64]. Although the primary endpoint was not achieved, the data suggest improved analgesia and a phase-3 trial is currently on-going (NCT02609828) (*level of evidence III, degree of recommendation C*).

### Psychological approaches to cancer pain

Psychological aspects such as emotional stress, anxiety, depression, uncertainty, and hopelessness influence the perception of pain and hinder its control [65]. Inversely, pain can cause or worsen psychological problems. This vicious circle must be broken by actions directed both at pain relief and patients’ psychological needs. Furthermore, we should treat the symptoms that often accompany pain: fatigue, sleep disorders, anorexia, etc., that impair patients’ quality of life and concern them.

Several meta-analyses and randomized clinical trials have shown that pain intensity can be reduced through psychological interventions (*level of evidence I, degree of recommendation A*). Cognitive-behavioral and mind-body therapies (relaxation, imagery, hypnosis, and biofeedback) can be extremely useful (*level of evidence II, degree of recommendation B*) [66]. Music, exercise, and yoga can help patients during cancer treatment, as well as cancer survivors [67].

Multidisciplinary care is essential and should include oncologists, psychologists, social workers, rehabilitators, etc. Equally necessary is good communication between the team caring for the patient and the patient’s family.

## Pain in cancer survivors

Up to 40% of cancer survivors present pain and in 5–10% it is both chronic and severe [68]. Strong opioids may be indicated but, since 40% of cancer survivors live longer than 10 years, there is a growing concern about the long-term side effects of opioids, opiate use to treat anxiety, overdose, and abuse in “non-patients”. Therefore, greater emphasis should be placed on non-opioid analgesics and non-pharmacological therapies.
